# Myosin VI contributes to maintaining epithelial barrier function

**DOI:** 10.1186/1423-0127-20-68

**Published:** 2013-09-13

**Authors:** Yu-Wei Liao, Xing-Mao Wu, Jia Jia, Xiao-Lei Wu, Tao Hong, Ling-Xin Meng, Xiu-Ying Wu

**Affiliations:** 1 Department of Anesthesiology, Shengjing Hospital, China Medical University, 36 Sanhao St, Shenyang 110004, China

**Keywords:** Endosome, Lysosome, Epithelium, Barrier function, Myosin

## Abstract

**Background:**

Epithelial barrier dysfunction is associated with the pathogenesis of a number of immune inflammations; the etiology is not fully understood. The fusion of endosome/lysosome is a critical process in the degradation of endocytic antigens in epithelial cells. Recent reports indicate that myosin VI (myo6) is involved in the activities of endosomes. The present study aims to investigate the role of myo6 in epithelial barrier dysfunction.

**Results:**

The endosome accumulation was observed in myo6-deficient Rmcs. More than 80% endosomes were fused with lysosomes in naïve Rmcs while less than 30% endosomes were fused with lysosomes in the myo6-deficient Rmcs. The myo6-deficient Rmc monolayers showed high permeability to a macromolecular antigen, ovalbumin, the latter still conserved the antigenicity, which induced strong T cell activation.

**Conclusions:**

We conclude that myo6 plays a critical role in the fusion of endosome/lysosome in Rmc epithelial cells. Deficiency of myo6 compromises the epithelial barrier function.

## Background

The epithelial barrier is formed by epithelial cell bodies and the tight junctions around the top of each epithelial cell. Such a barrier separates the body tissue from the external environment. Under the physiological states, the epithelial barrier only allows water and substances with small molecular weight to pass through [1]. Pathogens and macromolecular antigens are generally prevented from entering into the deep tissue by the epithelial barrier [2]. However, under the states of epithelial barrier dysfunction, the macromolecular antigens do enter the deep tissue in the body to interact with immune cells and induce skewed immune responses and cause immune inflammations [3,4]. The pathogenesis of epithelial barrier dysfunction is to be further understood.

Protein antigens can pass the dysfunctional epithelial barrier via paracellular pathways [5] or intracellular pathways [6] to enter the deep regions of tissue. The paracellular pathway is sealed by the tight junctions under normal physiological conditions [5]. In harsh conditions, such as psychological stress, the paracellular pathway may be opened to allow noxious substances to be absorbed [7]. Alternatively, antigens can be also transported across the epithelial barrier via the intracellular pathways [6]. In the process of via the intracellular pathways, the antigens are firstly wrapped by the plasma membranes to form endosomes, and then to be transported to the subepithelial region [8]. Theoretically, the endosomes can fuse with lysosomes within epithelial cells [9]. The endocytosed antigens should be degraded by the acidic hydrolases of lysosomes [9]. However, according to previous reports, the antigen-carrying endosomes can be transported across the epithelial barrier to be delivered into the deeper regions of tissue [10]. The phenomenon implies that the fusion of endosome and lysosome may be disturbed in the case of epithelial barrier dysfunction; the underlying mechanism needs to be further investigated.

Recent reports suggest that myosin VI (myo6) is involved in the activities of endosome and lysosome [11] in body cells. Myo6 belong to the myosin family that is composed by the ATP-dependent motor proteins, which play a role in muscle contraction and are involved in a wide range of other eukaryotic motility processes. After engulfing by epithelial cells, antigens can be wrapped to form endosomes. Previous studies observed that the endosomes in intestinal epithelial cells could move from the luminal side to the bottom side [10]. Thus, we hypothesized that myo6 may be involved in the movement of the endosomes. In this study, we knocked down the gene of myo6 from epithelial cells and found that the myo6-deficient epithelial cells showed endosome accumulation, less frequency of the fused endosome and lysosome and the endocytic protein antigens were transported across the epithelial barrier.

## Methods

### Reagents

The antibodies of EEA1, LAMP2, myosin VI and shRNA (sc-37133-SH) of myo6 were purchased from Santa Cruz Biotech (Shanghai, China). The ^51^Cr-EDTA was purchased from Chemindustry (Shanghai, China). Ovalbumin (OVA) and bovine serum albumin (BSA) were purchased from Sigma Aldrich (Shanghai, China). The ELISA kit of OVA was purchased from the AlphaDiagnosis (Hangzhou, China). Reagents of real time RT-PCR were purchased from Invitrogen China (Shanghai, China). The reagents of immune cell purification were purchased from Miltenyi Bioltech (Shanghai, China).

### Cell culture

The RPMI 2650 cells (Rmc, in short; a Human nasal epithelial cell line) were purchased from ATCC (Manassas, VA) and grown in Eagle’s minimal essential medium (MEM) supplemented with 100 U/ml penicillin, 100 μg/ml streptomycin and 10% fetal bovine serum (FBS) in a humidified incubator at 37°C with 5% CO2. The cells were further cultured in Transwells (polycarbonate membrane, 0.4 μm pore size, 1.12 cm2 surface area, Corning Costar Co., USA) to form monolayers to be used in further experiments.

### Permeability of Rmc monolayer assessment

Rmcs were cultured for 2 weeks in Transwell inserts to confluence (TER ≥500 Ohm/cm^2^). Two types of tracers were tested. For the macromolecular tracer, OVA (10 μg/ml) was added to the apical chambers of Transwells. Samples were taken from the basal chambers and analyzed the levels of OVA by ELISA. For the small molecular tracer, ^51^Cr-EDTA (Ethylenediaminetetraacetic acid; 1 μCi) was added to the apical chambers of Transwells. The [^51^Cr]- radioactivity was counted using a γ-counter. The permeability was expressed as the ratio between the original amount added to the apical chambers and the recovered amount from the basal chambers.

### Recording transepithelial resistance (TER)

The TER of Rmc monolayer was recorded using the Millicell ERS apparatus (Millipore, Bedford, Massachusetts, USA).

### Enzyme-linked immunoassay (ELISA)

The levels of OVA in the basal chambers of Transwells were determined by ELISA with commercial reagent kits following the manufacturer’s instruction.

### Western blotting

Total proteins were extracted from the cells. The protein extracts were fractioned in sodium dodecyl sulfate polyacrylamide gel electrophoresis and transferred onto nitrocellulose membrane. The membrane was incubated with primary antibodies (100 ng/ml) at 4°C overnight. After washing with TBST (Tris-buffered saline with Tween-20), the membrane was incubated with HRP-labeled secondary antibodies (50 ng/ml). The immune blots were developed with the ECL Western Blotting Reagent (Amersham; Shanghai, China).

### RNA interference of myo6

**Figure 1:**
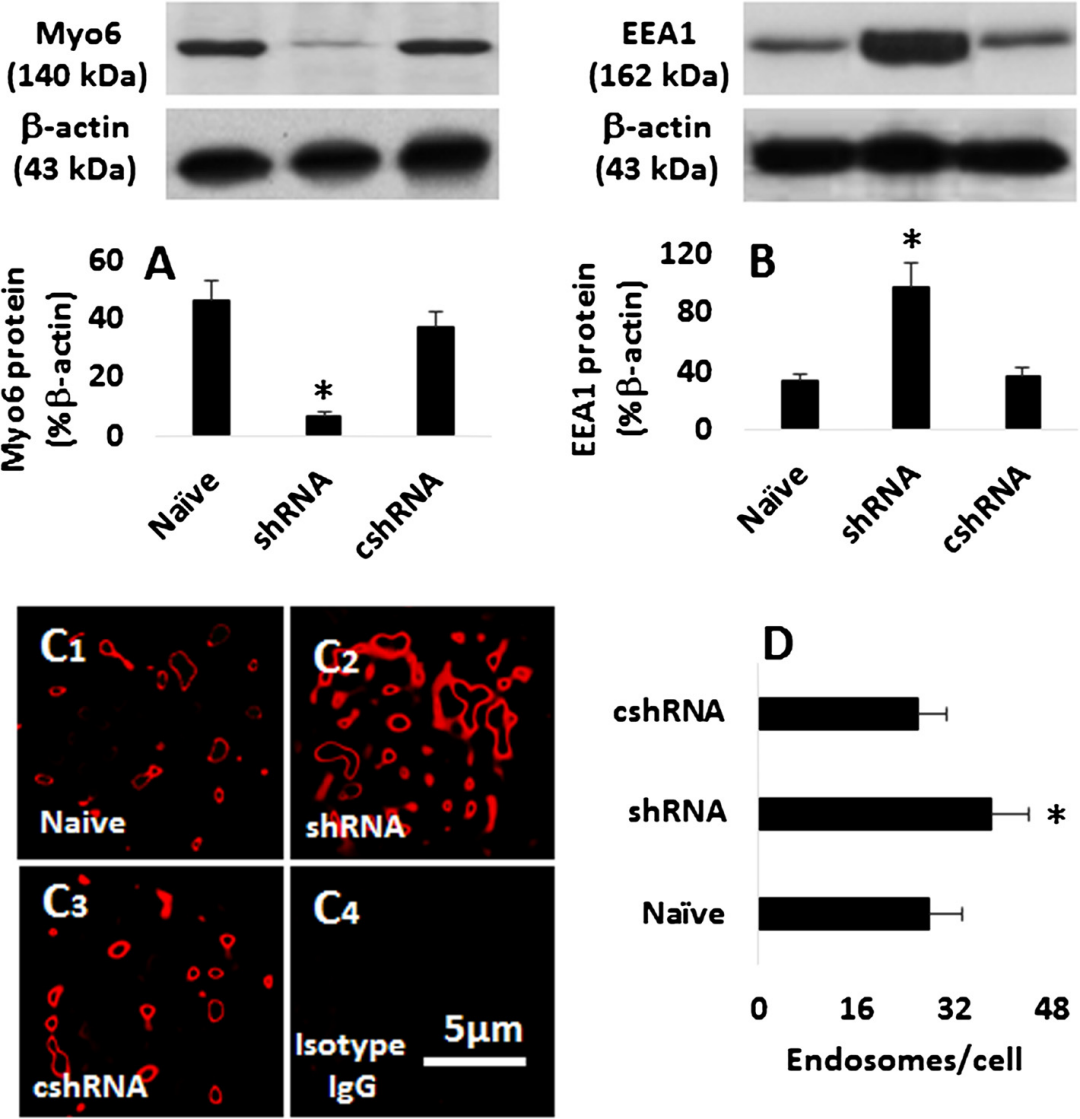
**Myo6 deficiency induces endosome accumulation in epithelial cells. A-B**, the immune blots indicate the levels of myo6 **(A)** or EEA1 (**B**; a marker of endosome) in epithelial cells. The bars below he blots indicate the summarized integrated density of the blots. **C**, the confocal images show the EEA1 staining (in red) in epithelial cells. **D**, the bars indicate the average number of endosomes in epithelial cells, which was averaged from 30 cells. Naïve: Naïve epithelial cells. shRNA: The epithelial cells were treated with shRNA of myo6. cshRNA: The epithelial cells were treated with control shRNA. The data in bar graphs were presented as mean ± SD. *, p < 0.01, compared with the naïve group. The data represent 3 separate experiments.

The myo6 gene was knocked down in Rmcs with the shRNA of the myo6 (non-specific shRNA was used as a control; cshRNA) following the manufacturer’s instruction. When the Rmcs in Transwells reach 3/4 confluent, the lentiviral vectors carrying the shRNA of myo6 or cshRNA were added to the inserts. The Rmc monolayers were kept culturing. After reaching confluence, the monolayers were used in the designed experiments. The gene knockdown effect was presented in Figure 1A, which occurred 48 h after the transduction. As we observed the effects in separate experiments, the gene knockdown effect lasted for at least for 2 weeks (data not shown).

### Immunocytochemistry

Following published procedures with modifications [12], endosomes and lysosomes were stained in Rmcs. The cells were fixed with 2% paraformaldehyde for 2 h at room temperature, incubated with the primary antibodies (0.5 μg/ml) for 1 h at room temperature and followed by incubating with fluorescence-labeled secondary antibodies (0.5 μg/ml) for 1 h at room temperature. The cells were smeared onto a slide and observed under a confocal microscope with the × 630 objective. When the positive staining of interest was identified, the images were further enlarged using the built-in enlarging software feature. Thirty images were photographed from experimental each group. The total number of positive stained particles in the cells was counted on each image. The OVA+/EEA1+ and OVA+/EEA1+/LAMP1+ particles were counted respectively.

### Preparation of immune cells

The CD3^+^ CD4^+^ CD25^-^ T cells (Th0) and dendritic cells (DC) were isolated from the spleen of OVA-TCR transgenic DO11.10 mice (purchased from Beijing Experimental Animal Center; Beijing, China) by the magnetic cell sorting with commercial reagent kits following the manufacturer’s instruction (the cell purity was more than 95% as checked by flow cytometry. The cells were cultured in RPMI1640 medium for further experiments. The using animal in the study was approved by the Animal Ethic Committee at our university.

### Antigenicity assay

The isolated Th0 cells were labeled with CFSE (Carboxyfluorescein succinimidyl ester) and cultured with DC (T cell: DC = 10:1; 10^5^ cells/ml) in the presence of OVA (10 μg/ml; BSA was used as an irrelevant control protein) or sample protein (from the basal chamber medium) in culture medium for 3 days. The CFSE-dilution assay was performed with a flow cytometer (FACSCanto, BD Bioscience, San Jose, CA).

### Statistical analysis

The data were presented as mean ± SD. Differences between two groups were determined by Student t test. p < 0.05 was set as the significant criterion.

## Results

### Deficiency of myo6 results in an accumulation of endosomes in epithelial cells

Published data indicate that the intracellular transport pathway plays a role in the pathogenesis of epithelial barrier dysfunction [13]. The process by which epithelial cells transport antigens across the epithelial barrier remains largely unknown. Recent studies indicate that the endosome/lysosome system and myo6 in epithelial cells plays a critical role in the transporting endocytic cargo [12,14,15]. Thus, we firstly observed the role of myo6 in the endosome metabolism in epithelial cells. We cultured an airway epithelial monolayer with or without the myo6 gene knockdown (Figure 1A). The epithelial cells were then processed for determining the levels of EEA1, a protein marker of endosome [16]. As shown by Western blotting, EEA1 protein was detected in naïve epithelial cells, which was markedly increased in myo6-deficient epithelial cells (Figure 1B). The results were confirmed by morphological study; the immunocytochemical staining showed much more non-fused endosomes and EEA1 staining in the myo6-deficient epithelial cells than that in control cells (Figure 1C-D). The results indicate that myo6 plays a critical role in the metabolism of endosomes in epithelial cells.

### Myo6 Facilitates fusion of endosome and lysosome

**Figure 2:**
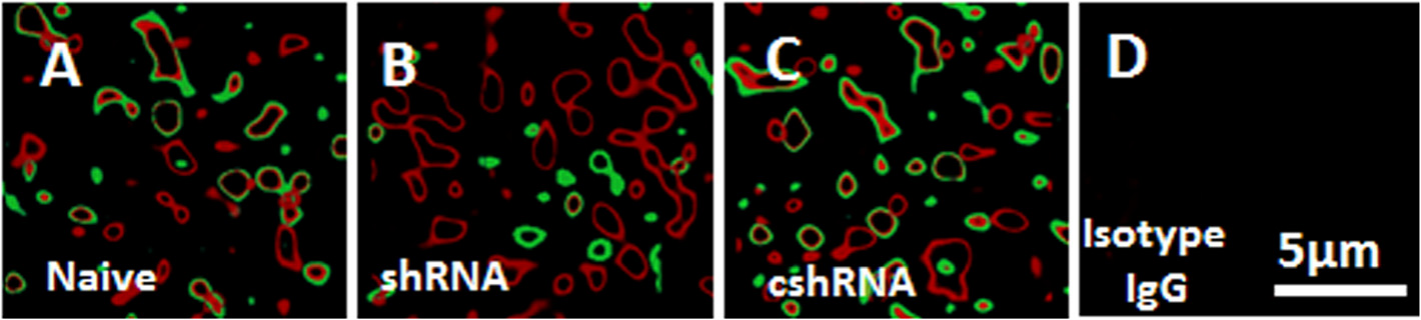
**Immune staining of endosome and lysosome.** The confocal images indicate the positive staining of EEA1 (in red) and LAMP2 (in blue) in epithelial cells. The treatment of the cells was annotated on each image. **A** (Naive), naïve epithelial cells. **B** (shRNA): The epithelial cells were treated with shRNA of myo6. **C** (cshRNA): The epithelial cells were treated with control shRNA. **D** (Isotype IgG): Cells were stained with isotype IgG using as a control. The data represent 3 separate experiments.

The fusion of endosome and lysosome is an important procedure in the metabolism of the endocytic cargo in phagocytes and epithelial cells. The factors regulate the fusion of endosome and lysosome have not fully understood yet. Since myo6 is associated with the activities of endosomes [11], we wondered if the deficiency of myo6 could affect the fusion of endosome and lysosome in epithelial cells. To this end, we further examined the cell samples of Figure 1 by immunocytochemistry with the cells stained with anti-EEA1 and LAMP2 (a marker of lysosome). As shown by confocal microscopy, the positive staining of EEA1, LAMP2 and the merge of EEA1 and LAMP2 appeared as granules in epithelial cells. We randomly selected 30 cells from each experimental group; the positively stained granules were counted. The results showed that in naïve epithelial cells (Figure 2A; Table 1), more than 90% EEA1^+^ positive granules were also LAMP2^+^ while the EEA1^+^ alone granules or LAMP2^+^ alone granules were less than 3% respectively. The results from myo6-deficient epithelial cells (Figure 2B; Table 1) were quite different from those myo6-sufficient epithelial cells. Among the myo6-deficient epithelial cells, only 3.2% EEA1^+^/LAMP2^+^ granules were observed; the EEA1^+^ granules were 68.7% and the LAMP2^+^ granules were 28.2%. The epithelial cells treated with control shRNA (Figure 2C; Table 1) showed similar results to naïve epithelial cells.

**Table 1:** Positively stained granules in epithelial cells

	**Total**	**EEA1+**	**LAMP2+**	**EEA1+/LAMP2+**
**Naïve**	895	72 (8.0%)	68 (7.6%)	755 (84.4%)
**shRNA**	856	352 (41.1%)*	284 (33.2%)*	220 (25.7%)*
**cshRNA**	903	85 (9.4%)	74 (8.2%)	744 (82.4%)

Naïve: Naïve epithelial cells. shRNA: The epithelial cells were treated with shRNA of myo6. cshRNA: The epithelial cells were treated with control shRNA. Isotype IgG: Cells were stained with isotype IgG using as a control. The data in Table 1 were presented as mean (SD). *, p<0.01, compared with naïve group. The data represent 3 separate experiments.

### Deficiency of myo6 promotes intracellular antigen transport across epithelial barrier

**Figure 3:**
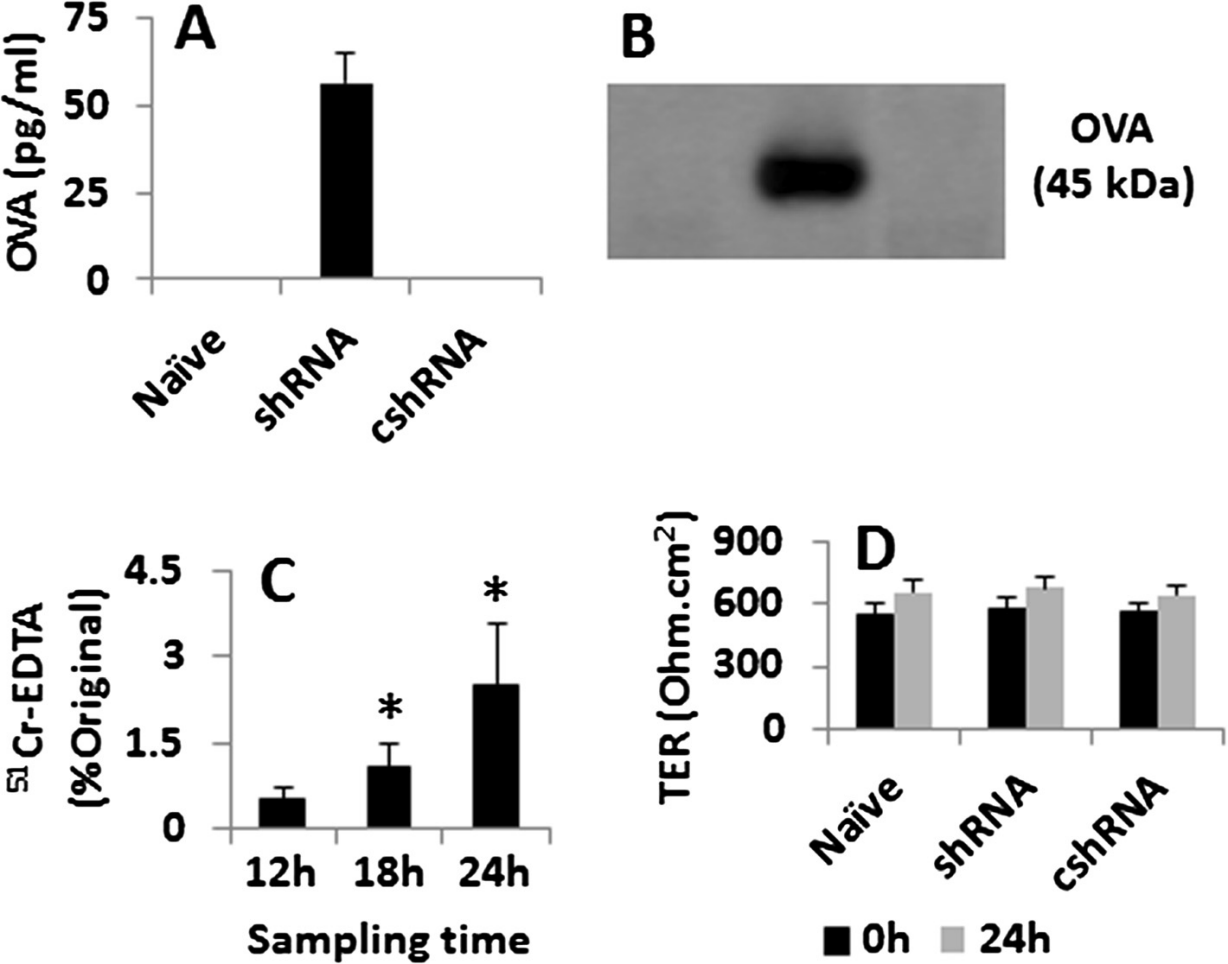
**Assessment of epithelial monolayer permeability. A**, the bars indicate the levels of OVA in basal chambers (assessed by ELISA). **B**, the immune blots indicate OVA in basal chambers (assessed by Western blotting). **C**, the bars indicate the levels of ^51^Cr-EDTA in basal chambers (assessed by a γ counter). **D**, the bars indicate the TER of the epithelial monolayers recorded at o h and 24 h respectively. The data in bar graphs were presented as mean ± SD. *, p < 0.05, compared with group 12 h **(C)** or naïve group **(D)**. Naïve: Naïve epithelial cells. shRNA: The epithelial cells were treated with shRNA of myo6. cshRNA: The epithelial cells were treated with control shRNA. The data represent 3 separate experiments.

The data of Figure 2 imply that the deficiency of myo6 disturbs the fusion of endosome and lysosome. Since the endocytic cargo, such as protein antigens, is degraded by the acidic hydrolases in lysosomes of epithelial cells, we wondered if the deficiency of myo6 affected the degradation of endocytic antigens in epithelial cells. To this end, we prepared epithelial cell monolayers with Transwells. After reaching confluence, OVA was added to the apical chambers. The samples were collected from the basal chambers 24 h later. As shown by the data of ELISA and Western blotting, no detectable OVA was transported across the monolayers. To clarify if the monolayers were permeable, we added a small molecular weight tracer, ^51^Cr-EDTA, to the apical chambers of Transwells. The samples from the basal chambers of Transwells showed that the monolayers were permeable to ^51^Cr-EDTA in a time-dependant manner. To assess the role of myo6 in the degradation of the endocytic antigens, we knocked down the gene of myo6 from epithelial cells. The myo6-deficient epithelial cells were cultured into monolayers. Samples were taken from the basal chambers 24 h later (Figure 3). The results showed a marked increase in the antigen, OVA, was detected in the basal chambers while the monolayers treated with control shRNA did not disturb the permeability of the monolayers. The results showed that myo6 plays an important role in the degradation of the endocytic antigens in epithelial cells.

### Antigens passed through the myo6-deficient epithelial monolayers conserve antigenicity

**Figure 4:**
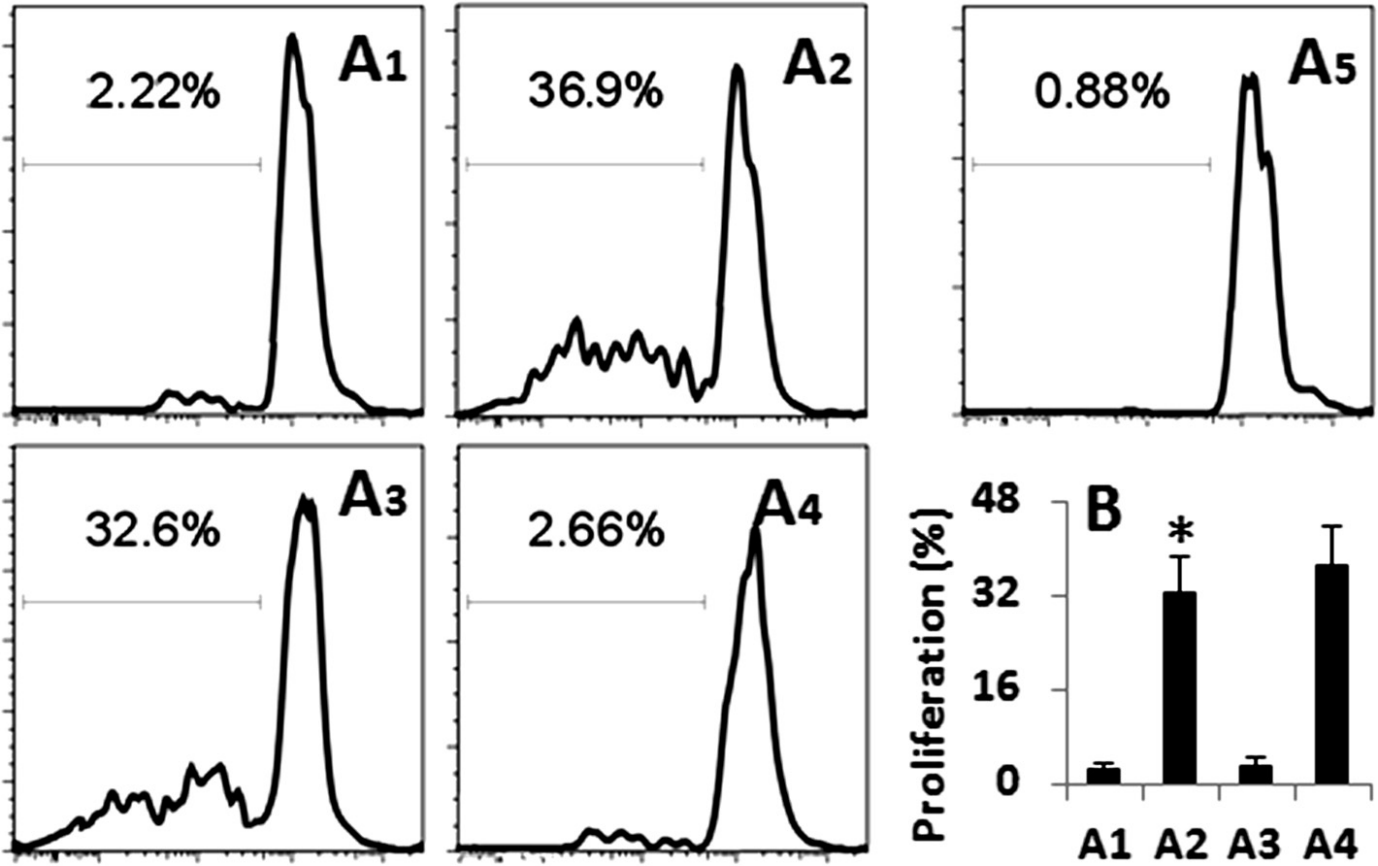
**T cell proliferation.** The T cell proliferation was assessed by CFSE-dilution assay. A1-A4, the histograms indicate the T cell proliferation. The numbers indicate the frequency of proliferating T cells. A5 is an isotype control. B, the bars indicate the summarized data of A1-A4. The data in the bar graph were presented as mean ± SD. *, p < 0.01, compared with group A1. The data represent 3 separate experiments.

To elucidate if the antigens passed through the myo6-deficient epithelial monolayers still conserved the antigenicity, we isolated the CD3^+^ CD4^+^ CD25^-^ Th0 cells from the OVA-TCR transgenic mouse spleen and cultured in the presence of dendritic cells and OVA (positive control) or samples extracted from the basal chambers of Transwells, or BSA (an irrelevant antigen using as a control) in the culture for 3 days. The T cell proliferation was assessed by the CFSE-dilution assay. The results showed that the Th0 cells did not proliferate with no specific stimuli (Figure 4A1, B). About 36.9% Th0 cells proliferated after stimulating by OVA (Figure 4A2, B). Samples taken from the basal chambers of Transwells of myo6-deficient epithelial monolayers caused 32.6% T cell proliferation (Figure 4A3, B) that was similar to that caused by OVA. Stimulated by BSA did not induce T cell proliferation (Figure 4A4, B).

## Discussion

In this study, we found that endosomes accumulated in the myo6-deficient epithelial cells. Myo6 was required in the process of the fusion of endosome and lysosome in epithelial cells. The knockdown of myo6 gene compromised the epithelial barrier function manifesting the increase in the permeability to macromolecular protein antigens. After passing the myo6-deficient epithelial barrier, the antigens still conserved the antigenicity.

The epithelial barrier function plays a critical role in the maintenance of the homeostasis of the body. The pathogenesis of a number of diseases, such as allergic diseases [17] and chronic immune inflammation [18], are associated with the epithelial barrier dysfunction. Various factors, such as psychological stress, microbial infection and radiations, are suggested associated with the epithelial barrier dysfunction. The present data have added a piece of novel information to this area; the myo6 plays a critical role in the maintenance of the epithelial barrier function. The data indicate that the myo6-deficiency-induced epithelial barrier hyperpermeability is via the intracellular pathway since the TER, the indicator of the paracellular functional state, is not altered in the epithelial monolayers. Myo6 is thought to transport endocytic vesicles into the cell, functions in a variety of intracellular processes such as vesicular membrane traffic, cell migration, and mitosis [19]. The present data indicate that myo6 is also involved in the fusion of endosome/lysosome. Inhibition of myo6 results in accumulation of endosomes in the cytoplasm as we observed in the present study. The phenomenon implicate that myo6 is required in the fusion of endosome/lysosome to render the endocytic cargo to be digested by the enzymes in lysosome. Shorting of myo6 may drive the endosomes with endocytic cargo, such as macromolecular antigens, to be transported to the subepithelial region to contact immune cells; some of the antigens may induce unwanted immune responses.

The sub-cellular pathology of this study showed that the myo6-deficient epithelial cells had the endosome accumulation. Under physiological states, the early endosomes form late endosomes, and then, fuse with lysosomes. The acidic hydrolases in lysosomes can degrade the endocytic cargo in endosomes. The accumulation of endosomes in the myo6-deficient epithelial cells implies the metabolism of the endosomes may be disturbed. The inference is supported by the subsequent data; much less endosome/lysosome fusion was observed in the myo6-deficient epithelial cells. The data are in line with others’ findings. Chen et al [20] reported that the ubiquitin TNFAIP3-deficient epithelial cells had less fusion of endosome/lysosome; the cells showed less ability to degrade the endocytic microbial products. Huang et al [21] suggested that ubiquitin myo6-deficient Caco-2 monolayers had disturbed fusion of endosome/lysosome.

The permeability is an important aspect to mirror the epithelial barrier function. In the present study, we observed marked increases in the permeability of the myo6-deficient epithelial monolayers; abundant protein antigens were transported across the epithelial monolayers to arrive the basal chambers of Transwells. The Transwell system is a mimic of epithelial barrier; the basal chambers are somewhat similar to the subepithelial region in the mucosa. The transported antigens have the opportunity to interact with immune cells in the subepithelial region. Thus, whether the antigens still conserve the antigenicity is of significance. Our further results corroborate the reasoning by showing that the transported antigens have strong antigenicity to induce T cell proliferation. Similar results were also reported recently; Song et al [14] found that antigens passed through the 20-deficient epithelial monolayers could activate antigen specific T cells.

## Conclusions

Taken together, the data indicate that the fusion of endosome/lysosome plays a critical role in the maintenance of the epithelial barrier function. The deficiency of myo6 can compromise the epithelial barrier function by increasing the intracellular permeability to macromolecular protein antigens.

## Competing interests

The authors declare that they have no competing interests.

## Authors’ contributions

YWL, XMW, JJ, XLW, TH and LXM performed experiments, analyzed the experimental data and reviewed the manuscript. XYW designed the project, supervised the experiments and wrote the paper. All authors read and approved the final manuscript.

## References

[B1] Goto Y Kiyono H Epithelial barrier: an interface for the cross-communication between gut flora and immune system Immunol Rev 2012 245 1 147 163 10.1111/j.1600-065X.2011.01078.x 22168418

[B2] Kinnebrew MA Pamer EG Innate immune signaling in defense against intestinal microbes Immunol Rev 2012 245 1 113 131 10.1111/j.1600-065X.2011.01081.x 22168416 PMC4624287

[B3] Wallon C Yang PC Keita V Ericson AC McKay DM Sherman PM Corticotropin-releasing hormone (CRH) regulates macromolecular permeability via mast cells in normal human colonic biopsies in vitro Gut 2008 57 1 50 58 17525093 10.1136/gut.2006.117549

[B4] Soderholm JD Yang P-C Ceponis P Vohra A Riddell R Sherman PM Chronic stress induces mast cell-dependent bacterial adherence and initiates mucosal inflammation in rat intestine Gastroenterology 2002 123 4 1099 1108 10.1053/gast.2002.36019 12360472

[B5] Ma Y Semba S Khan MRI Bochimoto H Watanabe T Fujiya M Focal adhesion kinase regulates intestinal epithelial barrier function via redistribution of tight junction Biochim Biophys Acta 2013 1832 1 151 159 10.1016/j.bbadis.2012.10.006 23064287

[B6] Takeuchi H Furuta N Morisaki I Amano A Exit of intracellular porphyromonas gingivalis from gingival epithelial cells is mediated by endocytic recycling pathway Cell Microbiol 2011 13 5 677 691 10.1111/j.1462-5822.2010.01564.x 21155963

[B7] Vicario M Guilarte M Alonso C Yang P Martínez C Ramos L Chronological assessment of mast cell-mediated gut dysfunction and mucosal inflammation in a rat model of chronic psychosocial stress Brain Behav Immun 2010 24 7 1166 1175 10.1016/j.bbi.2010.06.002 20600818

[B8] Yang PC Jury J Soderholm JD Sherman PM McKay DM Perdue MH Chronic psychological stress in rats induces intestinal sensitization to luminal antigens Am J Pathol 2006 168 1 104 114 10.2353/ajpath.2006.050575 16400013 PMC1592661

[B9] Takahashi Y Nada S Mori S Soma-Nagae T Oneyama C Okada M The late endosome/lysosome-anchored p18-mTORC1 pathway controls terminal maturation of lysosomes Biochem Biophys Res Commun 2012 417 4 1151 1157 10.1016/j.bbrc.2011.12.082 22227194

[B10] Berin MC Kiliaan AJ Yang PC Groot JA Taminiau JA Perdue MH Rapid transepithelial antigen transport in rat jejunum: impact of sensitization and the hypersensitivity reaction Gastroenterology 1997 113 3 856 864 10.1016/S0016-5085(97)70180-X 9287977

[B11] Bond LM Arden SD Kendrick-Jones J Buss F Sellers JR Dynamic exchange of myosin VI on endocytic structures J Biol Chem 2012 287 46 38637 38646 10.1074/jbc.M112.373969 22992744 PMC3493908

[B12] An YF Li TL Geng XR Yang G Zhao CQ Yang PC Ubiquitin E3 ligase A20 facilitates processing microbial product in nasal epithelial cells J Biol Chem 2012 287 42 35318 35323 10.1074/jbc.M112.392639 22936803 PMC3471685

[B13] Cameron HL Yang PC Perdue MH Glucagon-like peptide-2-enhanced barrier function reduces pathophysiology in a model of food allergy Am J Physiol Gastrointest Liver Physiol 2003 284 6 G905 G912 12736145 10.1152/ajpgi.00231.2002

[B14] Song CH Liu ZQ Huang S Zheng PY Yang PC Probiotics promote endocytic allergen degradation in gut epithelial cells Biochem Biophys Res Commun 2012 426 1 135 140 10.1016/j.bbrc.2012.08.051 22925894

[B15] Hegan PS Giral H Levi M Mooseker MS Myosin VI is required for maintenance of brush border structure, composition, and membrane trafficking functions in the intestinal epithelial cell Cytoskeleton 2012 69 4 235 251 10.1002/cm.21018 22328452 PMC3328626

[B16] Yokogawa M Kobashigawa Y Yoshida N Ogura K Harada K Inagaki F NMR analyses of the interaction between the FYVE domain of early endosome antigen 1 (EEA1) and phosphoinositide embedded in a lipid bilayer J Biol Chem 2012 287 42 34936 34945 10.1074/jbc.M112.398255 22915584 PMC3471759

[B17] Liu T Ma J Li TL Yang JF Liang X Yang PC High expression of CD98 alters epithelial barrier functions to promote induction of airway allergy Clin Exp Allergy 2012 42 7 1051 1059 10.1111/j.1365-2222.2012.03978.x 22702504

[B18] Hering NA Fromm M Schulzke JD Determinants of colonic barrier function in inflammatory bowel disease and potential therapeutics J Physiol 2012 590 5 1035 1044 22219336 10.1113/jphysiol.2011.224568 PMC3381811

[B19] Buss F Spudich G Kendrick-Jones JMYOSINVI Cellular functions and motor properties Annu Rev Cell Dev Biol 2004 20 1 649 676 10.1146/annurev.cellbio.20.012103.094243 15473855

[B20] Chen C Yang G Geng XR Wang X Liu Z Yang PC TNFAIP3 Facilitates degradation of microbial antigen SEB in enterocytes PLoS One 2012 7 9 e45941 10.1371/journal.pone.0045941 23029332 PMC3454357

[B21] Huang P GXYGCCLZYPC. Ubiquitin E3 ligase A20 contributes to maintaining epithelial barrier function Cell Physiol Biochem 2012 30 3 702 710 10.1159/000341450 22854680

